# Relationship between treatment delay and final infarct size in STEMI patients treated with abciximab and primary PCI

**DOI:** 10.1186/1471-2261-12-9

**Published:** 2012-02-23

**Authors:** Tim Tödt, Eva Maret, Joakim Alfredsson, Magnus Janzon, Jan Engvall, Eva Swahn

**Affiliations:** 1 Department of Medical and Health Sciences, Division of Cardiology, Linköping University, Linköping, Sweden, Department of Cardiology UHL, County Council of Östergötland, SE-581 85 Linköping, Sweden; 2 Department of Clinical Physiology, Ryhov County Hospital, SE-551 85 Jönköping, Sweden; 3 Center for Medical Image Science and Visualization, Linköping University, SE-581 83 Linköping, Sweden; 4 Department of Clinical Physiology UHL, County Council of Östergötland, SE-581 85 Linköping, Sweden

**Keywords:** Angioplasty, Balloon, Coronary, Emergency Medical Services, Myocardial Infarction, Myocardial Reperfusion, Time Factors, Time and Motion Studies

## Abstract

**Background:**

Studies on the impact of time to treatment on myocardial infarct size have yielded conflicting results. In this study of ST-Elevation Myocardial Infarction (STEMI) treated with primary percutaneous coronary intervention (PCI), we set out to investigate the relationship between the time from First Medical Contact (FMC) to the demonstration of an open infarct related artery (IRA) and final scar size.

Between February 2006 and September 2007, 89 STEMI patients treated with primary PCI were studied with contrast enhanced magnetic resonance imaging (ceMRI) 4 to 8 weeks after the infarction. Spearman correlation was computed for health care delay time (defined as time from FMC to PCI) and myocardial injury. Multiple linear regression was used to determine covariates independently associated with infarct size.

**Results:**

An occluded artery (Thrombolysis In Myocardial Infarction, TIMI flow 0-1 at initial angiogram) was seen in 56 patients (63%). The median FMC-to-patent artery was 89 minutes. There was a weak correlation between time from FMC-to-patent IRA and infarct size, r = 0.27, *p *= 0.01. In multiple regression analyses, LAD as the IRA, smoking and an occluded vessel at the first angiogram, but not delay time, correlated with infarct size.

**Conclusions:**

In patients with STEMI treated with primary PCI we found a weak correlation between health care delay time and infarct size. Other factors like anterior infarction, a patent artery pre-PCI and effects of reperfusion injury may have had greater influence on infarct size than time-to-treatment per se.

## Background

Reperfusion of the occluded artery in ST elevation myocardial infarction (STEMI) is preferably performed with primary Percutaneous Coronary Intervention (PCI) and adjunctive therapy with Glycoprotein (Gp) IIb/IIIa inhibitors even if this requires transportation of the patient to an institution that performs angioplasty [1-3]. Studies on the impact of time to treatment on mortality have revealed conflicting results [4-8]. Recently, Francone et.al studied 70 patients with STEMI with contrast enhanced Magnetic Resonance Imaging (ceMRI) and found that a shorter time to reperfusion was associated with smaller infarct size. This effect was mainly limited to patients treated within 90 minutes of symptom onset [9]. Brodie et al. found similar results when analysing patients from the EMERALD trial with a greater impact of the duration of ischemia in anterior versus non anterior infarction [10]. Haase et al. studied 45 STEMI patients and found no correlation between infarct size and time from onset of symptoms to PCI [11]. In a study comparing prehospital fibrinolysis with facilitated PCI there was a relationship between time to treatment, infarct size and transmurality [12]. Also Tarantini et al. found an association between time to treatment and the risk of a larger scar [13]. We hypothesised that in patients with STEMI treated with primary PCI and adjunctive abciximab, time from first medical contact (FMC) to demonstration of an open infarct related artery (IRA) is associated with the degree of myocardial injury measured with ceMRI.

## Methods

### STEMI strategy in the county of Östergötland

Since 2005 the Heart Centre of Östergötland, serving a population of 420 000 has adopted a strategy of primary PCI for all patients with STEMI, with prehospital administration of abciximab in suitable cases. Patients who present with symptoms of Myocardial Infarction (MI) and ST elevation or extensive anterior ST depression or bundle branch block on the ECG are sent to the cath-lab with the intention of performing primary PCI. Pretreatment with acetylsalicylic acid 300 mg orally is recommended in the absence of contraindications. Bolus abciximab (0.25 mg/kg), heparin (50 U/kg) and beta blockers are given at the discretion of the attending physician. Physicians are encouraged to give pretreatment with abciximab to patients with clear ST elevation on the diagnostic ECG and distinct symptoms of MI. Patients on warfarin, with a history of bleeding, of old age, or for other reasons having a high risk for bleeding, or patients without a clear diagnosis of STEMI are usually not given abciximab before arrival in the cath lab. If not given as pretreatment abciximab is given only after angiography has confirmed occlusion/stenoses suitable for PCI.

### Study population

Between February 2006 and September 2007, 149 STEMI patients treated with primary PCI at the Heart Centre of Östergötland and no known contraindication to ceMRI gave their written informed consent to participate in the study. Sixty patients were excluded due to one or more of the following reasons; previous myocardial infarction (7), death before ceMRI (3) reinfarction (1), new revascularisation with either CABG or PCI (15), new contraindication to ceMRI (3), lost to follow up (1), malignancy (1), claustrophobia while in the magnet (4), inability to perform the whole ceMRI study (2) and unwillingness to come back to the hospital to perform the ceMRI study (23). Thus, 89 patients remained for further analysis. In a broader picture the total number of primary PCI performed during the study period was 589. Thus, the majority of STEMI patients was not asked to participate either due to administrative reasons, long distance to the hospital, early transfer to the local hospital, early cardiac surgery or other factors preventing the participation of the patient. One year mortality was 12.7% in this group.

### Magnetic resonance imaging

ceMRI was performed 4-8 weeks after the date of primary PCI. The patients were placed in the magnet (1.5 T Achieva, Philips Healthcare, Best, the Netherlands) in supine position. A circular polarized body-array surface coil was used in all measurements. ECG-triggered MR images were obtained during repeated breath-holds.

**Figure 1:**
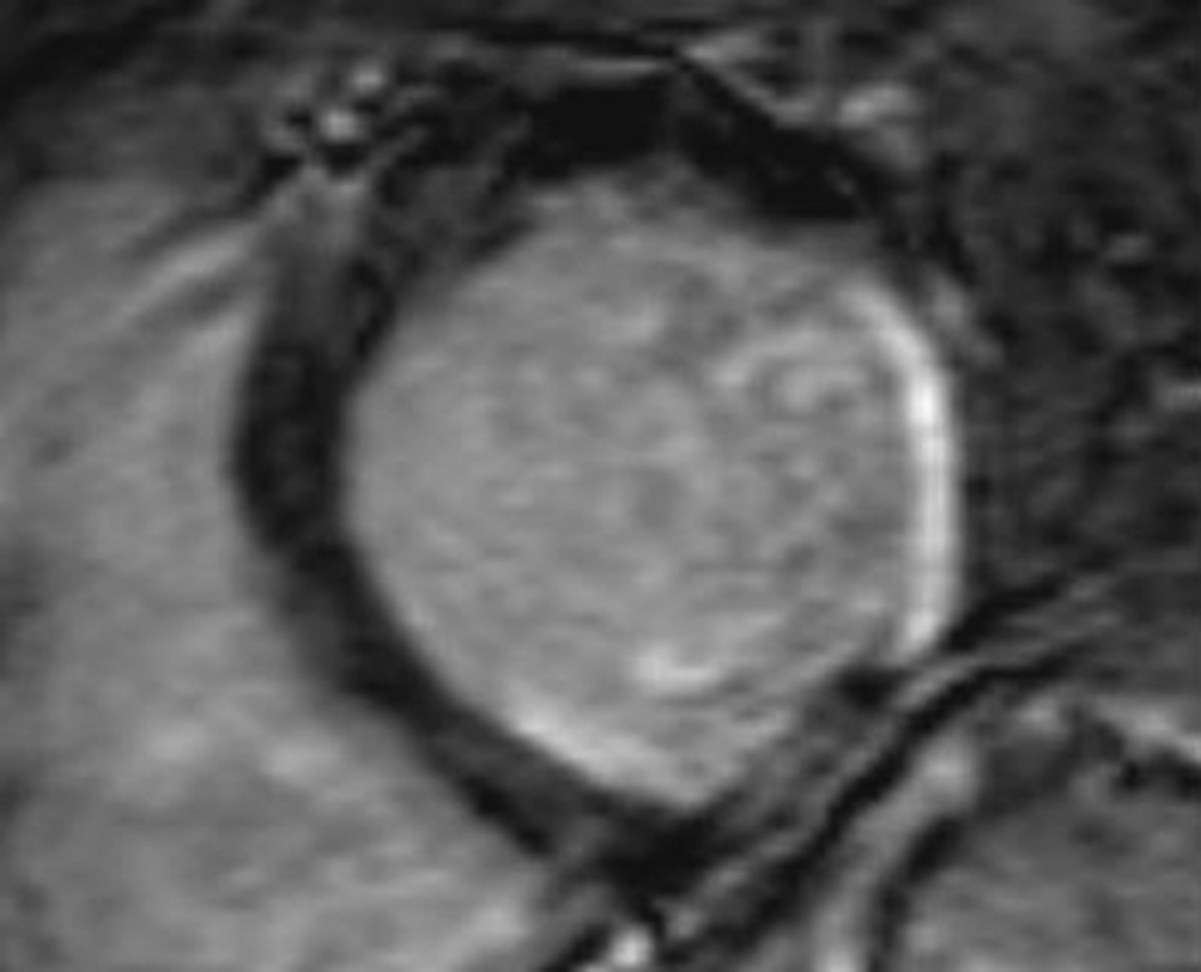
**Late gadolinium enhancement of lateral subendocardial scar in the shortaxis view of the left ventricle**. Same patient as in Additional files 1, 2, 3. The time from first medical contact (FMC) to patent artery in this particular patient was 105 minutes.

Cine-loops were acquired with a balanced steady state free precession turbo field-echo (b-SSFP TFE) sequence, on average 18, (range 10-23) short axis slices and three long axis planes (apical 2-, 3- and 4-chamber views). Temporal resolution was in between 26-41 ms (30 acquired phases). The inversion recovery turbo field echo (IR-TFE) sequence was a segmented 3D spoiled gradient echo sequence with TE = 1.3 ms, TR = 4.4 ms and TFE factor 43, leading to an acquisition phase time of 188 ms during diastole. Slice thickness was 10 mm intersection gap -5 mm (i.e. slices were overcontiguous), field-of-view 350 mm and image matrix 128 × 256. The contrast-enhanced images were acquired at the same slice positions as the cine-images, about 20 min after the administration of gadopentetate dimeglumine (Gd-DTPA) 0.2 mmol/kg bodyweight (Schering Nordiska AB, Järfälla, Sweden). Optimal contrast between hyperenhanced areas and normal myocardium was maintained by continually adjusting the inversion time to null the signal from healthy myocardium, Figure 1. Scar size was measured by two different observers on short-axis images using the freely available software "Segment" http://segment.heiberg.se[14]. Infarct volume and percentage was calculated from the short axis stack of slices. End diastolic myocardial and cavity volumes were measured from the short axis late gadolinium enhanced (LGE) images.

### Angiography

All angiograms were reviewed by three experienced PCI operators blinded for all other parts of the study. The following items were analysed: location of the acute vessel occlusion, Thrombolysis In Myocardial Infarction (TIMI) flow before and after intervention, type of PCI performed, success of treatment, and extent of coronary artery disease. Disagreement between observers was solved by consensus. The time when a patent Infarct Related Artery (IRA) was obtained was defined as the time of balloon inflation in patients with TIMI 0-1 flow at the first angiogram. In patients with TIMI 2-3 flow on arrival, the time of the first angiogram demonstrating an open culprit artery was considered as time of patent IRA. First Medical Contact (FMC) was defined as time when the patient called the ambulance system for patients admitted with ambulance directly to the cath lab and time for arrival to the Emergency Department for other patients. Patients were followed for one year for the occurrence of rehospitalisation for angina, myocardial infarction, PCI or CABG and for mortality.

The study protocol was approved by the Regional Ethical Review Board in Linköping and adhered to the Declaration of Helsinki.

### Statistical analysis

Data were analysed with SPSS software, version18.0 (SPSS,Chicago, Illinois). Continuous variables were reported as mean +/- SD or median (25th-75th percentile) as appropriate. For normally distributed variables two-tailed Student's *t*-test was used to verify differences between groups. Non-normally distributed variables were compared using the Mann- Whitney *U *test. Categorical variables were expressed as counts (percentage). Statistical significance was assessed with the Chi Square or the Fischer's Exact test. Spearman correlations were computed for time from FMC to an open IRA and ceMRI measurements of myocardial scar.

Multiple linear regression analysis was used to determine independent correlates of infarct size. Variables included were age, sex, current smoker, kidney function, diabetes, LAD as the culprit artery, TIMI 0-1 flow at the first angiogram and time from FMC to demonstration of a patent artery. Variables were entered simultaneously into the model. All reported p- values are two sided. *P*-values < 0.05 were considered statistically significant.

## Results

**Figure 2:**
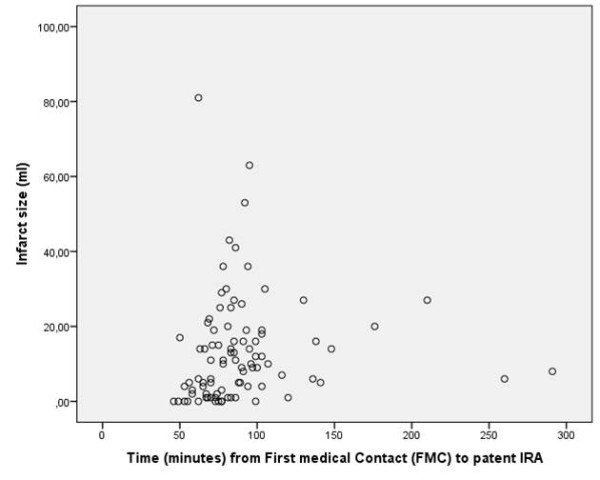
**Relationship between time (minutes) from first medical contact (FMC) to an open Infarct Related Artery (IRA) and absolute scar size (ml), r = 0.27, *p *= 0.01**.

Patient mean age was 62 years (SD 9.7). All patients had definite ST elevation on their diagnostic ECG and had elevated troponins. PCI was performed on a clearly visible culprit lesion and all patients were discharged with a diagnosis of myocardial infarction. A minority of patients (16%) were women. The median time from FMC to an open artery was 89 minutes. All but one patient received abciximab in conjunction with primary PCI of whom 79% were pre-treated with abciximab before arriving at the cath lab. A majority of patients were admitted directly to the cath lab by ambulance. No patient was lost to follow up (Table 1). The culprit vessel was the left anterior descending artery (LAD) in 46%, the circumflex artery (CX) in 12% and the right coronary artery (RCA) in 42% of patients. A patent artery (TIMI 2 or 3 flow) at the first angiogram was observed in 33 (37%) patients (Table 2). An example of an occluded circumflex artery at arrival is seen in Additional file 1, the flow effect of passing the guide wire in Additional file 2 and the final effect of balloon inflation and stent placement in Additional file 3. CeMRI was performed within 42 days (range 27-65) of the index infarction. There were no cardiovascular events between the index myocardial infarction and ceMRI (19 of the 149 patients were excluded due to this criterion). Time from FMC to a patent IRA correlated weakly with absolute infarct size measured with ceMRI (Figure 2). Patients with a patent artery within 90 minutes after FMC appeared to have smaller infarct size in absolute and relative terms than those with longer delay times although this did not reach statistical significance (Table 3). Using multiple regression analysis, LAD as a culprit vessel.

**Table 1:** Baseline characteristics

	FMC to patent artery < 90 min (N = 48)	FMC to patent artery ≥ 90 min (N = 41)	*P *value
Age, years (SD)	61.8 (9.7)	62.9 (9.8)	0.62

Women	5 (10.4)	9 (22.0)	0.14

**Risk factors**			

Diabetes Mellitus	4 (8.3)	1 (2.4)	0.37

Hypertension	12 (25.0)	13 (31.7)	0.48

Current smoker	21 (43.8)	18 (43.9)	0.99

Hyperlipidemia	1 (2.1)	3 (7.3)	0.33

Previous Revascularization	0 (0)	0 (0)	

Creatinine clearance ml/min (SD)	81 (23.1)	74 (18.6)	0.60

**Clinical presentation**			

Systolic blood pressure, mmHg (SD)	145 (29.6)	136 (26.4)	0.12

Heart rate, beats/min (SD)	70 (14)	66 (14)	0.18

Presentation off hours	27 (56.3)	32 (78.0)	0.03

Admission with ambulance	44 (91.7)	34 (82.9)	0.21

Pretreatment with abciximab	37 (77.1)	33 (80.5)	0.70

**Time delays, median, minutes (IQR)**			

Symptom to FMC	91 (51-191)	90 (28-229)	0.43

FMC to angiography	66 (58-79)	93 (85-116)	0.0005

FMC to patent artery	78 (68-83)	107 (100-131)	0.0005

FMC to balloon	84 (76-100)	111 (104-140)	0.0005

Symptom to abciximab	150 (80-195)	160 (78-285)	0.48

Abciximab to PCI	53 (33-68)	63 (43-78)	0.13

Data are presented as counts (percentage) if not otherwise indicated. SD Standard Deviation. IQR Interquartile range. FMC, First Medical Contact. Creatinine clearance calculated Cockroft Gault formula ((140 - Age) * (Wt in kg) * (0.85 if female)/(72 * cr) (Plasma Creatinine * 0.8136)). PCI Percutaneous coronary intervention

**Table 2:** Angiographic data

	FMC to patent artery < 90 min (N = 48)	FMC to patent artery ≥ 90 min (N = 41)	*P *value
**Extent of coronary disease**			

One vessel disease	25 (52.1)	22 (53.7)	0.88

Two vessel disease	15 (31.3)	12 (29.3)	0.84

Three vessel disease	9 (18.8)	6 (14.6)	0.60

**Infarct related artery**			

LAD/Diagonal	24 (50.0)	17 (41.5)	0.42

Cx/Intermediate/Marginal	3 (6.3)	8 (19.5)	0.058

RCA/RPD/Posterolateral	21 (43.8)	16 (39.0)	0.65

**TIMI flow at first angiogram**			

TIMI 0	20 (41.7)	26 (63.4)	0.04

TIMI 1	5 (10.4)	5 (12.5)	1.0

TIMI 2	12 (25.0)	4 (9.8)	0.06

TIMI 3	11 (22.9)	6 (14.6)	0.32

Data are presented as counts (percentage). FMC - First Medical Contact. LAD - Left anterior descending artery. Cx - Circumflex artery. RCA - Right coronary artery. RPD - Right posterior descending artery. TIMI - Thrombolysis in Myocardial Infarction

**Table 3:** Cardiac Magnetic Resonance data

	FMC to patent artery < 90 min (N = 48)	FMC to patent artery ≥90 min (N = 41)	*P *value
Scar%	4 (1-14)	8 (4-13)	0.06

Scar ml	6 (1-18)	12 (6-19)	0.07

Data are presented as median with interquartile range. FMC, First Medical Contact

Active smoking and an occluded vessel (TIMI 0-1) at the first angiogram, but not time from FMC to demonstration of a patent artery, correlated with infarct size (Table 4). During one year follow up there was no mortality. A new myocardial infarction occurred in two patients and a new revascularisation with PCI and/or CABG was performed in 16 (18%) patients. In patients with an occluded IRA at presentation in the cath lab (n = 56) there was no correlation between FMC to an open vessel (r = 0.13, *p *= 0.36) and infarct size. There were 10 patients with no visible scar on ceMRI. These patients were admitted due to symptoms of an acute coronary syndrome with ST elevation on their diagnostic ECG. They had a clear culprit lesion treated with primary PCI that corresponded to the ECG findings. The peak troponin in this group of patients was 3.5 (0.4-14) ug/l, (cut off value 0.05 ug/l) and they were discharged from the initial hospital stay with a diagnosis of myocardial infarction. The patients without detectable scar were not regarded as aborted infarctions and were included in the total analysis. Patients with no scar on ceMRI six weeks after infarction were younger, tended to have less extensive coronary disease, had shorter time from FMC to a patent artery and had a TIMI 2-3 flow at presentation in 80% of cases.

**Table 4:** Multiple linear regression.

	B (95% CI)	*P *value
**Variable**		

Age	0.99 (-23-22)	0.96

Occluded artery at first angiogram	11 (5-17)	0.0005

LAD as culprit artery	11 (5-17)	0.0005

FMC to patent artery (minutes)	0.02 (-0.06-0.09)	0.61

Active Smoker	7 (1-13)	0.02

Male sex	4 (-3-12)	0.26

eGFR	-0.1 (-0.3-0.1)	0.23

Diabetes	1 (-10-13)	0.82

Predictors of infarct size (Scar ml)

B, regression coefficient. CI, confidence interval. FMC - First Medical Contact. LAD, Left anterior descending artery. eGFR,estimated glomerular filtration rate

## Discussion

In this single centre prospective study of patients with STEMI we found a weak correlation between First Medical Contact (FMC) to an open IRA (median time 89 minutes) and infarct size measured with ceMRI. Measurements of overall treatment delay could be hampered by recall bias and measurement error. Therefore, in the main analysis we have used the robust measure of time from FMC-to-patent artery [15] instead of symptom-to-patent artery. However the relationship for total delay time and infarct size was only marginally weaker (r = 0.21, *p *= 0.05) than health care delay time and infarct size (r = 0.27, *p *= 0.01). Even if the analysis was restricted to patients with an occluded artery at presentation, i.e. TIMI 0-1 flow, we found no correlation between time from symptom onset, (r = 0.12, *p *= 0.38), or FMC to a patent IRA (r = 0.13, *p *= 0.36) and infarct size. Patients who demonstrated a patent artery within 90 minutes of FMC showed a trend towards smaller infarct size than those who presented with longer delay times. Moreover, among 10 patients with no infarct detectable on ceMRI all but one had a FMC-to-patent artery time less than 80 minutes. Several studies have shown a correlation between time to treatment and mortality [16-18] as well as between time to treatment and myocardial salvage and damage measured with Single Photon Emission Computed Tomography (SPECT) or ceMRI [19,20]. Intuitively, we expect an association between time delay and scar size. Several mechanisms that dilute this association may be in operation and need to be accounted for, such as variable effects of reperfusion injury which may be responsible for as much as 25% of final infarct size [21]. The circumstances governing effects of reperfusion are only partly known [22]. In a pooled analysis of four contemporary primary PCI trials, Stone et al. showed longer symptom-onset-to-balloon time to be associated with larger infarct size measured with SPECT. Specifically there was a benefit in patients reperfused within three hours of coronary occlusion. In patients with PCI less than two hours after symptom onset infarct size was very small, being only 4% of the left ventricular myocardium [23]. These results are in line with a study by Hedström et al. who provided evidence that infarct size increased with the duration of ischemia in their population with an occluded artery [24]. There are several reasons why the correlation between time from FMC to open artery and scar size was weak in our study. A substantial number (37%) showed TIMI 2-3 flow in the IRA on the first angiogram [25]. The true time to spontaneous opening of the IRA is most likely shorter than the time attributed to those patients in the study. This fact may dilute the relationship between time delay and scar size. In patients with TIMI 2-3 flow at angiograpy, 82% received abciximab already in the ambulance. Moreover, these patients had significantly smaller infarct size measured on ceMRI (7 vs. 17 ml, *p *< 0.001) and 8 out of 33 patients with a patent IRA on arrival had no sign of myocardial scar at all. The benefit of a normal epicardial blood flow in the IRA before PCI has been documented in numerous studies [26-30]. Antegrade flow in the IRA has been associated with smaller infarct size [23,31,32] and could weaken the effect of longer health care delay time. The median time from symptom-to-angiography in our study was189 minutes which also is the median time for the whole population of STEMI patients treated with primary PCI in Sweden [33]. The total time delay to primary PCI in our population was 193 minutes. As shown in the study by Stone [23] and supported by experimental results [34] there is less effect on infarct size when PCI is performed two to three hours after coronary occlusion. Moreover, Francone et.al showed that myocardial salvage was detectable mainly in patients reperfused within 90 minutes after symptom onset [9] which was achieved in only three of our patients. Additionally, the median infarct size was small, 7% of LV mass, probably reflecting that the study population represented a healthier part of the wider spectrum of STEMI patients which reduces the possibility to demonstrate significant differences between groups based on the time delay to opening the vessel. Lastly, we cannot rule out that in a larger study population we would have seen a stronger correlation between time to treatment and infarct size.

### The influence of the infarct related artery

Patients with infarctions within the LAD territory had significantly shorter time from symptom onset to FMC (60 vs 130, *p *= 0.01) but no difference in FMC to PCI or a patent artery. (88 vs 89, *p *= 0.44). This could be interpreted in terms of a larger ischemic area producing more intense symptoms prompting patients to seek medical attention. The patients with LAD as the culprit artery actually developed larger infarct size, 16 ml vs. 9 ml, *p *= 0.02, despite shorter time to treatment [35,36]. Multiple regression analyses revealed LAD as the culprit artery, active smoking and an occluded vessel at the first angiogram to correlate with infarct size. This could be due to several factors such as, a) the LAD perfuses a larger myocardial territory than other vessels [37], b) the scar size from a maintained occlusion with a late reperfusion injury could be larger than from a shorter ischemic time combined with an early reperfusion, c) active smoking could be related to more advanced atherosclerosis, or to a hypercoagulable state lowering the chance of spontaneous thrombolysis [38].

### Limitations of the study

Only patients who survived the initial hospital stay and were free from a cardiac event during the interim period were included. CeMRI was performed on average six weeks after myocardial infarction and not in the early phase of infarction. However, studies have shown that infarct size measured with ceMRI is largest in the acute phase and diminishes gradually [39]. After 10 days the contrast enhanced area seems to be quite stable and allows reproducible measurement of the size of the final myocardial damage even if later shrinkage of scar is possible [40]. The median symptom-to-patent artery time was more than three hours and only three patients were treated within 90 minutes of symptom onset, which is the new gold standard proposed by the study by Francone et al. [9]. However the time delays in our study are not longer than measured in many other trials [23]. In addition, for patients with an open vessel at angiography, the time to open artery was taken as the time of the angiography, even if spontaneous opening of the vessel may have occurred earlier.

## Conclusions

In STEMI patients treated with primary PCI we found a weak correlation between FMC to demonstration of an open IRA and myocardial injury on ceMRI six weeks later. In this study, other factors like anterior infarction, a patent artery at the initial angiogram and effects of reperfusion injury seem to have greater influence on infarct size than time to treatment per se.

## Competing interests

The authors declare that they have no competing interests.

## Authors' contributions

TT, JA, MJ, JE and ES have been involved in conception and design. JE and ES recruited the subjects. TT, EM and JE have been involved in data acquisition. TT, EM and JE post processed the data. TT, JA, MJ, EM, JE and ES analysed and interpreted the data. TT performed the statistical analysis. TT, JA, MJ, JE and ES supervised the study. TT, JE and ES drafted the manuscript. TT, JA, MJ, EM, JE and ES critically revised the manuscript. All authors had full access to the data and take responsibility for its integrity. All authors have read and agree to the manuscript as written.

## Funding

This work was supported by the Medical Research Council of Southeast Sweden, the medical faculty of Linköping University, the Swedish Heart-Lung foundation, the Swedish Research Council, Futurum - the Academy for health Care Jönköping County Council and the Center of Medical Image Science and Visualization at Linköping University Hospital

## Pre-publication history

The pre-publication history for this paper can be accessed here:

http://www.biomedcentral.com/1471-2261/12/9/prepub

## Supplementary Material

Additional file 1 **Coronary angiography of STEMI-patient in **Figure 1. Initial angiogram shows occlusion of left circumflex coronary artery. Click here for file

Additional file 2 **Coronary angiography of STEMI-patient in **Figure 1. The guide wire has traversed the occlusion. Click here for file

Additional file 3 **Coronary angiography of STEMI-patient in **Figure 1. Final result after balloon inflation and placement of stent. TIMI flow III was achieved. Click here for file
